# Intermittent application of external positive pressure helps to preserve organ viability during ex vivo perfusion and culture

**DOI:** 10.1007/s10047-019-01141-3

**Published:** 2019-10-15

**Authors:** Kazunori Sano, Jun Homma, Hidekazu Sekine, Eiji Kobayashi, Tatsuya Shimizu

**Affiliations:** 1 Institute of Advanced Biomedical Engineering and Science, Tokyo Women’s Medical University, Tokyo, Japan; 2 Tokaihit Co., Ltd., Shizuoka, Japan; 3 Department of Organ Fabrication, Keio University School of Medicine, Tokyo, Japan

**Keywords:** Organ culture, Tissue engineering, Bioreactor, External intermittent pressure

## Abstract

The perfusion of medium through blood vessels allows the preservation of donor organs and culture of bioengineered organs. However, tissue damage due to inadequate perfusion remains a problem. We evaluated whether intermittent external pressurization would improve the perfusion and viability of organs in culture. A bioreactor system was used to perfuse and culture rat small intestine and femoral muscle preparations. Intermittent positive external pressure (10 mmHg) was applied for 20 s at intervals of 20 s. Intermittent pressurization resulted in uniform perfusion of small intestine preparations and minimal tissue damage after 20 h of perfusion, whereas non-pressurized (control) preparations exhibited significantly worse perfusion of the upper surface than the lower surface and histologic evidence of tissue damage. Longer term studies were undertaken in luciferase-expressing rat femoral muscle preparations. Compared with non-pressurized controls, intermittent pressurization led to better perfusion throughout the 14-day experimental period, improved organ viability as indicated by a higher bioluminescence intensity after perfusion with luciferin, and reduced levels of tissue necrosis with better preservation of vascular structures and skeletal muscle nuclei (histologic analyses). Therefore, intermittent application of external positive pressure improved the perfusion of small intestine and skeletal muscle preparations and enhanced tissue viability when compared with controls. We anticipate that this innovative perfusion technique could be used to improve the preservation of donor organs and culture of bioengineered organs.

**Electronic supplementary material:**

The online version of this article (10.1007/s10047-019-01141-3) contains supplementary material, which is available to authorized users.

## Introduction

The perfusion of medium through blood vessels facilitates the preservation of donor organs ex vivo and culture of bioengineered organs. Current transplantation strategies typically store a donor organ at 4 °C in an organ-preservation solution such as Belzer UW^®^ solution (Preservation Solutions Inc., Elkhorn, WI, USA), Custodiol^®^ HTK solution (Essential Pharmaceuticals, LLC, Newtown, PA, USA), or Euro-Collins solution (Kyowa CritiCare Co., Ltd, Tokyo, Japan). However, static cryopreservation techniques maintain organ viability for only a limited time [1]. Techniques for ex vivo perfusion of blood vessels have been developed to help preserve organs prior to transplantation. For example, successful transplantation was achieved after ex vivo normothermic perfusion of donor heart [2] and lungs [3].

Perfusion systems are commonly used in the field of regenerative medicine to construct thick tissues and organs, and vascularization of bioengineered tissue can achieve long-term survival of the tissue [4]. Cardiac tissue containing functional blood vessels has been bioengineered by layering cardiac cell sheets on a femoral vascular bed that was perfused with medium in a bioreactor system [5]. Furthermore, bioartificial heart, kidney, liver, and lung have been generated by a technique involving whole organ decellularization, recellularization with target cells and perfusion through pre-existing blood vessels [6–9]. Thus, perfusion systems are becoming increasingly useful for the preservation of donor organs and culture of bioengineered tissues.

Previous studies have compared pulsatile and constant flow for the perfusion of vascular structures, especially in the setting of kidney preservation [10]. Compared with constant flow, pulsatile flow was found to decrease peripheral vascular resistance and improve kidney preservation [11]. We hypothesized that applying a pulsatile external physical load would improve ex vivo organ perfusion and culture. Therefore, the aim of this study was to evaluate whether the perfusion and viability of organs in culture would be enhanced by the application of intermittent external pressurization. To achieve this aim, we cultured rat small intestine and skeletal muscle preparations in a custom-made perfusion bioreactor with a gas-driven external pressurizing device.

## Materials and methods

Detailed methods are provided in the Supplementary Data.

### Animals and study design

For the small intestine perfusion experiments, 8 Lewis rats (8–12 weeks; male; Charles River Laboratories Japan, Inc., Kanagawa, Japan) were divided into a pressurized group (*n* = 4) and control group (*n* = 4). The skeletal muscle perfusion experiments utilized 4 transgenic Lewis rats (LEW-Tg[Rosa-luc]11jmsk; 8–12 weeks; male) expressing firefly luciferase (Luc) [12]. Femoral muscle preparations from the left and right sides of each rat were used in separate experiments, enabling the following experimental grouping: pressurized group (*n* = 4) and control group (*n* = 4).

### Ethical approval

All animal experiments were approved by the Ethics Committee for Animal Experimentation of Tokyo Women’s Medical University and performed according to the Guidelines of Tokyo Women’s Medical University on Animal Use.

### Perfusion bioreactor system with intermittently applied external pressure

**Fig. 1:**
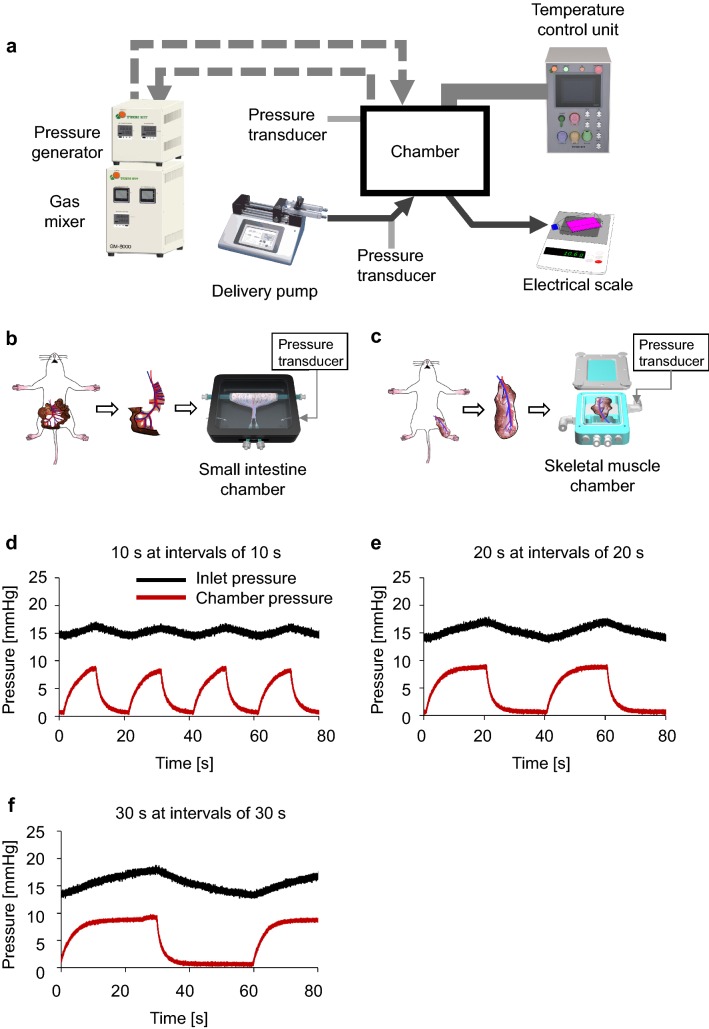
Intermittently pressurizing bioreactor and changes in chamber pressure and arterial inlet pressure of the small intestine preparation. **a** Schematic of the intermittently pressurizing bioreactor. The bioreactor consisted of an organ culture chamber that was connected to a pressure generator fed by a gas mixer, a syringe pump for delivery of medium, a temperature control unit, and a culture flask on an electronic balance for measuring the mass of the perfused medium collected from the venous outlet. The pressure transducers were connected directly to the chamber and the inlet perfusion pathway. **b** Sealed chamber used for perfusion of the rat small intestine preparation. **c** Sealed chamber used for perfusion of the rat skeletal muscle preparation. **d**–**f** Traces show variations in arterial inlet pressure (black) and chamber pressure (red) during the application of intermittent external pressure. **d** External positive pressure of 10 mmHg applied for 10 s at intervals of 10 s. **e** External positive pressure of 10 mmHg applied for 20 s at intervals of 20 s. **f** External positive pressure of 10 mmHg applied for 30 s at intervals of 30 s

The harvested organs were cultured ex vivo in a pressurizing bioreactor system (Tokaihit Co., Ltd., Shizuoka, Japan) consisting of a gas mixer, pressure generator, medium delivery pumps, temperature-conditioning unit, tube set, organ culture chamber, and electronic balance (Fig. 1a). Organ culture chambers were prepared individually for the small intestine (Fig. 1b) and skeletal muscle (Fig. 1c). The gas mixer was connected to the organ chamber and injected gas. The pressure generator was connected to the organ chamber via a pressure-adjustable path and a release path, allowing external pressure to be applied to the organ intermittently during perfusion and culture. The amount of perfused medium leaving the organ via its major vein was collected and measured continuously by the electronic balance.

### Short-term perfusion of small intestine preparations

Rats were anesthetized with 2–3% inhaled isoflurane and administered 400 IU/kg heparin sodium intraperitoneally (Mochida Pharmaceutical Co., Ltd., Tokyo, Japan). The terminal ileum (7–10 cm) with an intact superior mesenteric artery and vein was harvested. The small intestine preparation was placed in the culture chamber in the bioreactor system, and the artery, vein, and intestinal lumen were connected to the appropriate tubes. The small intestinal lumen was perfused with phosphate-buffered saline (PBS; 50 µL/min). The small intestinal vasculature was perfused with medium via the artery (50 µL/min) for 20 h at 37 °C.

### Long-term perfusion of skeletal muscle preparations

Rat femoral muscle with an intact artery and vein was surgically removed and set up for organ culture in the bioreactor system as described previously [5]. The skeletal muscle was perfused with medium (50 μL/min) for 14 days at 37 °C.

### Perfusion ratio analysis

The mass of medium leaving the tissue vein was recorded continuously by an electronic balance. The perfusion ratio (%) was calculated as: [mass of medium leaving the venous outlet tube (g)]/[mass of medium infused via the arterial inlet tube (g)] × 100.

### Measurement of vascular length in the blood-perfused small intestine

The small intestine was perfused with blood and fixed, and its upper and lower aspects were imaged using a stereomicroscopy system (MVX10 and cellSens Dimension, Olympus, Tokyo, Japan). Blood-perfused vessels in the images were traced, and their lengths relative to the area of the small intestine were quantified using the AngioTool software (National Institutes of Health, Bethesda, MD, USA). The surface area of the small intestine preparation was quantified using ImageJ 1.51j8 (National Institutes of Health, Bethesda, MD, USA). The tissue distribution of the perfused blood was analyzed separately for the upper and lower surfaces of the small intestine by calculation of the ratio of vascular length-to-surface area.

### Bioluminescence imaging of the perfused skeletal muscle preparation

Bioluminescence imaging was performed to assess skeletal muscle viability after 14 days of perfusion, as previously described [5]. During imaging, the preparation was continuously perfused with 0.1% d-luciferin firefly potassium salt (Promega, Madison, WI, USA; 50 μL/min), and intermittent external pressurization was applied continuously. To compensate for differences in the sizes of the skeletal muscle constructs, relative bioluminescence was calculated as the ratio of the value on day 1 to that on day 14.

### Histologic analyses

The organs were fixed with 4% paraformaldehyde (Wako Pure Chemicals, Osaka, Japan) and routinely processed into 10-μm-thick paraffin-embedded sections. The sections were stained with Heidenhain’s Azan trichrome stain or hematoxylin–eosin (HE) in accordance with standard protocols. The stained sections were imaged under a light microscope (Eclipse E800, Nikon, Tokyo, Japan; BZ-9000, Keyence, Osaka, Japan).

### Statistical analyses

Data were analyzed using SPSS 23 (IBM Corp., Armonk, NY, USA). All data were normally distributed (Kolmogorov–Smirnov test). Perfusion ratios are expressed as mean ± standard error and were compared between groups using one-way analysis of variance and Tukey’s post-hoc test. Blood-perfused vascular length ratios are presented as box-plots displaying median, interquartile range, and range and were compared between groups using the Tukey test. Relative bioluminescence values are presented as box-plot diagrams and were compared between the control and pressurized groups using the unpaired Student’s *t* test. A *p* value < 0.05 was considered statistically significant.

## Results

### Changes in chamber pressure and arterial inlet pressure induced by intermittent pressurization of the small intestine preparation

The perfused small intestine preparation was used to examine the effects of intermittent external pressurization on chamber pressure and arterial inlet pressure. External positive pressures of 10 mmHg were applied for 10, 20, or 30 s at intervals of 10, 20, or 30 s, respectively. Both the chamber pressure and arterial inlet pressure responded to changes in external pressure (Fig. 1d–f). The increase in chamber pressure reached a plateau at 10 mm Hg when the external pressure was applied for 20 s at intervals of 20 s (Fig. 1e) or for 30 s at intervals of 30 s (Fig. 1f). However, the chamber pressure did not plateau at 10 mmHg when the external pressure was given for 10 s at intervals of 10 s (Fig. 1d). Therefore, in all subsequent experiments, external pressure was applied for 20 s at intervals of 20 s. The arterial inlet pressure increased more gradually than chamber pressure during the application of external pressure. Furthermore, arterial inlet pressure failed to reach 10 mmHg in all cases (Fig. 1d–f).

### Perfusion ratio and blood-perfused vascular length in the small intestine preparation

**Fig. 2:**
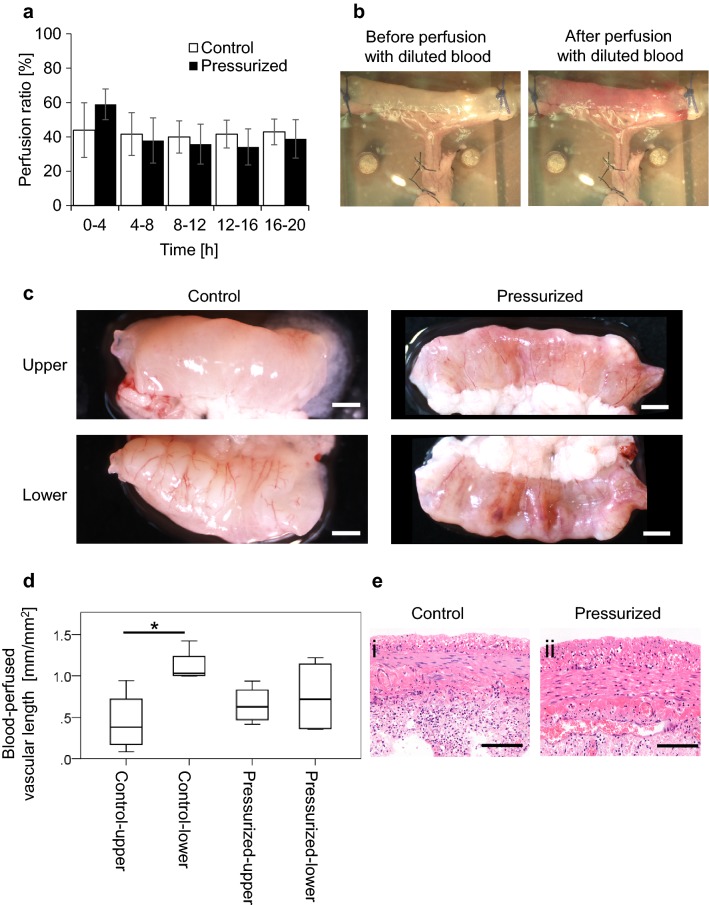
Effect of intermittent external pressurization on perfusion of the small intestine preparation. **a** Perfusion ratio of the small intestine preparation measured every 4 h over a period of 20 h. **b** After 20 h of perfusion and culture, the small intestine was perfused with diluted blood to enable visualization of perfusable blood vessels. The representative example shows a macroscopic image of a small intestine preparation before and after perfusion with diluted blood. **c** Representative examples from the control and pressurized groups showing the distribution of blood in the upper and lower surfaces of the small intestine after perfusion with diluted blood. Scale bars: 1 mm. **d** Box plots comparing blood-perfused vascular length between the upper and lower surfaces of small intestine preparations from the control and pressurized groups. The total length of blood-perfused vascular vessels visualized after perfusion with diluted blood was normalized to the surface area of the small intestine preparation (mm^−1^). *p < 0.05, *n* = 4. **e** Histologic images (stained with hematoxylin–eosin) of small intestine sections obtained after perfusion and culture for 20 h. Representative examples are shown from the control (i) and pressurized (ii) groups. Scale bars: 100 µm

Tissue perfusion of the small intestine preparation was evaluated by determination of the perfusion ratio every 4 h. The mean value for the entire 20 h period was similar between the pressurized group (57.3 ± 12.2%) and control group (42.5 ± 9.9%), and no significant differences between groups were detected at any timepoints (Fig. 2a).

Blood-perfused vascular length analysis was performed to evaluate the distribution of blood flow within the small intestine after 20 h of perfusion (Fig. 2b). In the control group, the blood vessel distribution pattern differed between the upper and lower surfaces of the small intestine, with the lower surface more homogeneously perfused than the upper surface. However, there was little difference in blood vessel distribution between the upper and lower surfaces in the pressurized group (Fig. 2c). Blood-perfused vascular length analysis showed that average vessel length was significantly shorter for the upper surface than for the lower surface in the control group (*p* < 0.05, *n* = 4). By contrast, blood-perfused vascular length did not differ significantly between the upper and lower surfaces of the small intestine in the pressurized group (Fig. 2d), indicating that vascular flow is more uniformly distributed when perfusion of medium is accompanied by intermittent external pressurization. Moreover, histologic analysis of the small intestine after 20 h of perfusion revealed less deterioration of tissue structure in the pressurized group than in the control group (Fig. 2e).

### Perfusion ratio and organ viability in the skeletal muscle preparation

**Fig. 3:**
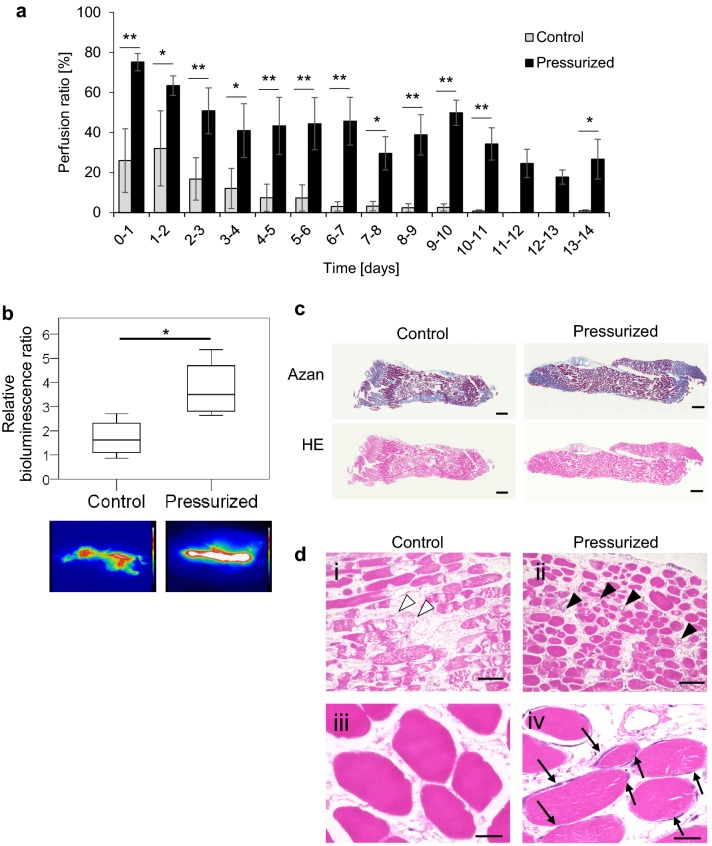
Effect of intermittent external pressurization on perfusion of the skeletal muscle preparation. **a** Perfusion ratio was measured during perfusion of rat femoral muscle for 14 days. The data are shown as mean ± standard error of the mean (*p < 0.05, **p < 0.01; *n* = 4). **b** Bioluminescence imaging assay. Relative bioluminescence was calculated as the ratio of the value on day 1 to that on day 14. Pseudo-color processing was employed to illustrate the differences in bioluminescence signal intensity. (*p < 0.05, *n* = 4). **c** Representative histologic images showing skeletal muscle sections stained with Azan or hematoxylin–eosin. Scale bars: 1 mm. **d** High-magnification images of skeletal muscle sections stained with hematoxylin–eosin. Degradation of vascular structures and loss of muscle cell nuclei were evident in specimens from the control group (i and iii), whereas vascular structures and muscle cell nuclei were preserved in specimens from the pressurized group (ii and iv). Scale bars: 200 µm (i and ii) and 50 µm (iii and iv). White arrowheads: degraded vascular structures. Black arrowheads: preserved vascular structures. Black arrows: muscle nuclei

To evaluate the longer-term effects of intermittent pressurization in a thicker organ than the small intestine, rat femoral muscle was perfused and cultured for 14 days. Skeletal muscle perfusion was assessed by calculation of the perfusion ratio every 24 h. The averaged perfusion ratio in the pressurized and control groups was 63.1 ± 6.3% and 24.9 ± 15.0%, respectively, for the first 3 days of perfusion/culture (*n* = 4) and 41.7 ± 6.8% and 8.0 ± 5.2%, respectively, over the entire 14-day period (*n* = 4). Notably, the perfusion ratio was significantly higher in the pressurized group than in the control group at every timepoint (*p* < 0.05, *n* = 4; Fig. 3a).

Bioluminescence imaging was used to assess the viability of skeletal muscle from luciferase-expressing rats. After 14 days of perfusion/culture, the bioluminescence intensity was significantly higher in the pressurized group than in the control group (*p* < 0.05, *n* = 4; Fig. 3b). These findings suggest that intermittent external pressurization improved the circulation of perfusate and helped to maintain the viability of rat femoral muscle during long-term culture.

### Histologic changes in the skeletal muscle preparation

After 14 days of perfusion/culture, histologic analysis (Azan and HE) of the femoral muscle preparation revealed more tissue necrosis in the control group than in the pressurized group (Fig. 3c). Moreover, degradation of vascular structures and loss of muscle cell nuclei were observed in the control group, whereas vascular structure and cell nuclei were preserved in the pressurized group (Fig. 3d).

## Discussion

Normothermic perfusion has been applied in several organ-preservation studies such as those involving the heart [13], liver [14], kidney [15], and lung [16]. Furthermore, successful transplantation has been performed after ex vivo normothermic perfusion of organs for 12 h [13, 16] or 20 h [15]. However, to our knowledge, no previous investigations have reported longer term (i.e., > 24 h) organ preservation with normothermic perfusion. In the present study, we evaluated whether a newly developed bioreactor system that intermittently applied external positive pressure would have a beneficial effect on the viability of organs perfused under normothermic conditions. We found that intermittent application of external pressure generated pulsatile flow in the vascular system of the small intestine. Furthermore, intermittent positive pressure improved the uniformity of perfusion in small intestine preparations and the perfusion ratio in skeletal muscle preparations. In addition, intermittent positive pressure helped to preserve vascular structures and surrounding tissue. Therefore, our novel findings demonstrate that long-term organ preservation can be achieved successfully by the use of intermittent pressurization to generate pulsatile vascular flow during normothermic perfusion.

Pulsatile flow has been reported to have several advantages over constant flow in ex vivo studies of the isolated kidney, including positive effects on vascular endothelial function (such as greater NO release from endothelial cells), blood vessel function, and renal viability (indicated by a higher renal oxygen demand) [17]. However, the pulsatile flow created by the heart in vivo is attenuated in the peripheral vascular system [18]. Therefore, when organs are cultured ex vivo, it is possible that pulsatile flow generated by an infusion pump might also be attenuated from the proximal end to the distal end of the inlet tube. In this study, pulsatile flow was generated by intermittent external positive pressurization in a bioreactor system. Pulsatile flow of medium to the entire vascular system, including peripheral blood vessels, was successfully achieved and led to long-term organ preservation (i.e., 14 days).

A pressure of 10 mmHg was utilized in the present study, because it is likely within the physiologic range of external pressures for the organs used. For example, intraperitoneal pressure in vivo can vary widely from 0.7 to 14 mmHg depending on body position and activity [19], and intramuscular pressure can range from 5.7 to 12 mmHg during the resting phase [20]. The pressure within the blood vessels of the small intestine varied with intermittent pressurization, indicating that the external pressure changes in our system affected not only the organ surface, but also its vascular system. Furthermore, when compared with no pressurization, intermittent external pressurization resulted in more uniform perfusion of the small intestine preparation and a better perfusion ratio in the skeletal muscle preparation. We speculate that intermittent compression of the microvascular system may improve the perfusion of medium through the vascular system by reducing stagnation of flow.

In this study, the external pressure changes were achieved using a gas-driven pressurizing bioreactor designed and built in-house. The pulsatile pressure generator had a pressure regulating path that determined the upper limit of pressure inside the organ chamber and a gas release path that enabled depressurization of the chamber. The chamber was sealed within a confined dead space, allowing the bioreactor system to accurately pressurize the organ to a predefined level by injecting gas into the chamber at a slow flow rate (i.e., 150–200 mL/min). Our experiments confirmed that the inner chamber pressure was reproducibly pressurized to 10 mmHg within 20 s and depressurized to 0 mmHg (relative to atmospheric pressure) within 20 s.

The perfusion ratio was defined as the ratio of the mass of medium leaving the organ via its vein to that of the medium infused via the artery. During ex vivo perfusion, some of the perfused medium leaks from the vascular system into the surrounding tissues and is lost from the surface of the organ. Previous studies have reported perfusion ratios of 40 ± 7% in skeletal muscle preparations perfused for 3 days [5] and 55.4 ± 4.4% in engineered skin flaps perfused for 3 days [21]. In our study, the perfusion ratio in the non-pressurized (control) group was 24.9 ± 15.0% over the course of 3 days, which is somewhat lower than the values obtained by the investigations mentioned above. One possible reason for this discrepancy is that our perfusion medium did not contain growth factors, whereas the previous studies included fibroblast growth factor-2 (FGF-2) in the medium perfusing skeletal muscle [5] and epidermal growth factor (EGF) in the medium used for skin flap perfusion [21]. FGF-2 induces the reorganization of endothelial junctions and deposition of extracellular matrix [22], and EGF modulates vascular tone and tissue homeostasis [23]. Since these growth factors were included to maintain the viability of the vascular system, it is reasonable to assume that they would increase the perfusion ratio by reducing the loss of medium from the vasculature. Although the medium that we used did not contain any growth factors, it was notable that the perfusion ratio in the pressurized group (63.1 ± 6.3%) was higher than the values reported by previous studies [5, 21]. Indeed, the perfusion ratio for the skeletal muscle preparation was substantially higher in the pressurized group than in the control group at day 3 (63.1 ± 6.3% vs. 24.9 ± 15.0%) and day 14 (41.7 ± 6.8% vs. 8.0 ± 5.2%). One likely mechanism underlying the differences between groups is that the vascular structures in the organ showed substantial deterioration in the non-pressurized group, but were relatively well maintained in the pressurized group. Consistent with our observations, von Horn et al. reported that pulsatile flow helped to sustain vascular function, reduce the leakage of medium, and maintain the perfusion ratio in the isolated perfused kidney [17]. In addition, it is likely that the smaller difference between internal vascular pressure and chamber pressure in the pressurized group suppressed the leakage of medium from the vessels to the surrounding tissue spaces.

The present study used bioluminescence imaging after perfusion with luciferin to evaluate the viability of femoral muscle harvested from luciferase-expressing rats [12]. After 14 days of ex vivo perfusion and culture, the bioluminescence intensity was observed to be higher in the pressurized group than in the control group, suggesting that organ viability had been better maintained in the pressurized group. This conclusion was supported by the findings of the histology experiments, which revealed less necrosis and better maintenance of vascular structures and muscle cell nuclei in the pressurized group. It is likely that maintenance of vascular structures in the pressurized group helped to sustain adequate levels of nutrient provision and waste product removal, thereby preserving cell viability.

Although it was beyond the scope of the present study to investigate the mechanisms of intermittent external pressurization at the cellular and molecular level, the previous research has yielded some insight into the effects of increased pressure and mechanical stimulation on various cell types. Ando et al. reported that shear stress promoted the differentiation of immature endothelial cells to mature endothelial cells that secreted factors such as NO [24, 25]. Jeong et al. found that vascular smooth muscle cells stimulated mechanically by pulsatile flow differentiated into a mature phenotype [26]. Wann et al. observed that an elevation in hydrostatic pressure increased the duration of the action potential in excitable cells by slowing both the peak depolarization and repolarization rates [27]. In addition, Wong et al. reported that cyclic hydrostatic pressure affected chondrocyte gene expression, slowed cartilage differentiation, and exerted an anti-angiogenic effect [28]. However, the relevance of these previously published findings to the present study is unclear, because much higher pressures were used than in our experiments (7600 to 76000-fold higher in the study of Wann et al. and 3800-fold higher in the study of Wong et al.). Notably, in our study, the perfusion ratio for the skeletal muscle preparation was significantly higher in the pressurized group than in the control group at day 0–1. This short-term outcome is more likely to be due to the effects of physical pressure at the organ level rather than direct actions at the cellular level involving changes in cell membrane properties due to altered gene expression. Therefore, as noted earlier, we speculate that the main beneficial effects of intermittent external pressurization on organ preservation are achieved through two mechanisms that cooperate to maintain the viability of cells in the tissue. First, intermittent external pressurization generates pulsatile flow of medium to the entire vascular system of the organ without attenuation of the pulsatile effect in peripheral vessels, and the pulsatile flow contributes to less stagnation of medium flow. Second, intermittent physical compression of the microvascular system may reduce the leakage of medium from the vessels to the tissue spaces. Nevertheless, it remains possible that low pressures such as those used in the present study might act directly on cells to affect their long-term viability. Further mechanobiological research will be needed to elucidate the mechanisms that contributed to the effects of intermittent pressurization observed in the present study.

This study has a limitation that should be noted. Our objective was to investigate whether the intermittent application of external positive pressure within the physiological range might have beneficial effects during ex vivo organ perfusion and culture, but our proof-of-concept study only utilized a pressure of 10 mmHg. It is possible that a pressure of 10 mmHg may not have been optimal and that more effective organ preservation might be achieved by the application of a different pressure. Further research utilizing a range of pressures will be needed to optimize the conditions for intermittent application of external positive pressure. Nonetheless, the data described in this manuscript provide strong evidence that the intermittently pressurized bioreactor system could potentially be used to facilitate the long-term preservation of donor organs or maturation of bioengineered tissues or organs.

## Conclusion

Intermittent application of external positive pressure using the bioreactor system described in this study improved the perfusion of small intestine and skeletal muscle preparations and enhanced tissue viability, as compared with no external pressurization. We anticipate that this innovative perfusion technique could be used to improve the preservation of donor organs and culture of bioengineered organs.

## Electronic supplementary material

Below is the link to the electronic supplementary material.

Supplementary file1 (DOC 64 kb)
