# Correction to: Intermittent application of external positive pressure helps to preserve organ viability during ex vivo perfusion and culture

**DOI:** 10.1007/s10047-019-01145-z

**Published:** 2019-11-07

**Authors:** Kazunori Sano, Jun Homma, Hidekazu Sekine, Eiji Kobayashi, Tatsuya Shimizu

**Affiliations:** 1 Institute of Advanced Biomedical Engineering and Science, Tokyo Women’s Medical University, Tokyo, Japan; 2 Tokaihit Co., Ltd., Shizuoka, Japan; 3 Department of Organ Fabrication, Keio University School of Medicine, Tokyo, Japan

## Correction to: Journal of Artificial Organs 10.1007/s10047-019-01141-3

The article Intermittent application of external positive pressure helps to preserve organ viability during ex vivo perfusion and culture, written by Kazunori Sano, Jun Homma, Hidekazu Sekine, Eiji Kobayashi and Tatsuya Shimizu, was originally published electronically on the publisher’s internet portal on 15 October 2019 without open access. With the author(s)’ decision to opt for Open Choice the copyright of the article changed on 15 November 2019 to © The Author(s) 2019 and the article is forthwith distributed under a Creative Commons Attribution 4.0 International License (https://creativecommons.org/licenses/by/4.0/), which permits use, sharing, adaptation, distribution and reproduction in any medium or format, as long as you give appropriate credit to the original author(s) and the source, provide a link to the Creative Commons licence, and indicate if changes were made.

The original article has been corrected.

