# Supplementary material for: Intermittent application of external positive pressure helps to preserve organ viability during ex vivo perfusion and culture
Source: J Artif Organs. 2019 Oct 15;23(1):36–45. doi: 10.1007/s10047-019-01141-3 (PMC7046602; doi:10.1007/s10047-019-01141-3)
Supplement: Supplementary file 1 — Supplementary file1 (DOC 64 kb) [file 10047_2019_1141_MOESM1_ESM.doc]

**Intermittent application of external positive pressure helps to preserve organ viability during *ex vivo* perfusion and culture**

Kazunori Sano, M.Sc.^1,2^, Jun Homma, M.D., Ph.D.^1^, Hidekazu Sekine, Ph.D.^1^, Eiji Kobayashi, M.D., Ph.D.^3^, Tatsuya Shimizu, M.D., Ph.D.^1^

^1^Institute of Advanced Biomedical Engineering and Science, Tokyo Women’s Medical University, Tokyo, Japan

^2^Tokaihit Co., Ltd., Shizuoka, Japan

^3^Department of Organ Fabrication, Keio University School of Medicine, Tokyo, Japan

**Corresponding author:**

Hidekazu Sekine

Institute of Advanced Biomedical Engineering and Science, Tokyo Women’s Medical University, Tokyo, Japan

Tel: +81-3-3353-8111　Fax: +81-3-3359-6046

E-mail: sekine.hidekazu@twmu.ac.jp

Supplementary method

**The perfusion medium and pressurizing gas for the small intestine perfusion culture**

The medium consisted of DMEM/M199 Hanks' (mixture Dulbecco's Modified Eagles Medium and Medium199 with Hanks' salts, Kohjin Bio Co. Ltd., Saitama, Japan) supplemented with 10% fetal bovine serum (Japan Bio Serum Co. Ltd, Hiroshima, Japan), 1% penicillin–streptomycin (Life Technologies, Rockville, MD, USA), 0.02 μg/mL sodium nitroprusside (Maruishi Pharmaceutical. Co. Ltd, Osaka, Japan) and 0.06 µg/mL amphotericin B (Nacalai Tesque Inc., Kyoto, Japan). Room air (obtained from the gas mixer) was used as the pressurizing gas when DMEM/M199 Hanks' was utilized as the culture medium. Before discontinuation of small intestine perfusion, the intestinal vessels were perfused with rat blood diluted 1:4 in PBS at a flow rate of 50 µL/min for 1 h.

**Preparation for long-term perfusion of skeletal muscle**

For the skeletal muscle perfusion experiments, rat femoral muscle with an intact artery and vein was surgically removed as described previously [6]. Briefly, the rat femoral muscle was partially resected from peripheral blood vessels using a thermal cautery unit (TCU-150, Bioresearch Center, Nagoya, Japan), and the skin incision was immediately closed. One week after the procedure, the rats were administered 400 IU/kg heparin intravenously, and the femoral muscle with the femoral artery and vein was resected. The rats were anesthetized with 2–3% inhaled isoflurane throughout all surgical procedures. After the blood had been washed out with PBS, the skeletal muscle preparation was placed in the organ culture chamber in the bioreactor system, and the artery and vein were connected to the respective inlet/outlet tubes.

**The perfusion medium and pressurizing gas for the skeletal muscle perfusion culture**

The skeletal muscle was perfused with DMEM (Sigma-Aldrich, St. Louis, MO, USA) containing 10% fetal bovine serum, 0.02 μg/mL sodium nitroprusside and 1% penicillin–streptomycin at a flow rate of 50 μL/min for 14 days at 37ºC. The perfusion medium was maintained at pH 7.4 by the provision of 5% CO_2_ from the gas mixer into the medium reservoir and organ culture chamber.

**Measurement of external pressure and flow pressure**

The arterial inlet pressure was monitored with a pressure transducer (MP5200, Edwards Lifesciences, Tokyo, Japan) that connected directly to the inlet perfusion pathway (Fig. 1a). The pressure of the chamber was monitored with a pressure transducer (MP5200, Edwards Lifesciences) that connected directly with the chamber (Fig. 1a). Before each experiment, the pressure transmitters were calibrated to read 0 mmHg at atmospheric pressure. The data were acquired using a PowerLab™ system (AD Instruments, New South Wales, Australia). For experiments utilizing intermittent external pressurization, the chamber pressure during the non-pressurization state was set at 0 mmHg by the pressure generator. The chamber pressure of the control group was maintained at 0 mmHg by release to the atmosphere.

**Bioluminescence imaging of the perfused skeletal muscle preparation**

Bioluminescence imaging was performed to assess skeletal muscle viability after 14 days of perfusion, as previously described [6]. A custom-made electron-multiplying CCD camera system and AquaCosmos 2.6 software (ImageEM, Hamamatsu Photonics, Shizuoka, Japan) were used for real-time bioluminescence imaging. During imaging of the skeletal muscle in the bioreactor system, the preparation was continuously perfused with 0.1% D-luciferin firefly potassium salt (Promega, Madison, WI, USA) at a flow rate of 50 μL/min, and intermittent external pressurization was applied continuously. The photon intensity of the skeletal muscle was expressed as total flux (photons/s). To compensate for differences in the sizes of the skeletal muscle constructs, relative bioluminescence was calculated as the ratio of the value on day 1 to that on day 14.
